# Secreted Cerberus1 as a Marker for Quantification of Definitive Endoderm Differentiation of the Pluripotent Stem Cells

**DOI:** 10.1371/journal.pone.0064291

**Published:** 2013-05-22

**Authors:** Hidefumi Iwashita, Nobuaki Shiraki, Daisuke Sakano, Takashi Ikegami, Masanobu Shiga, Kazuhiko Kume, Shoen Kume

**Affiliations:** 1 Department of Stem Cell Biology, Institute of Molecular Embryology and Genetics, Kumamoto University, Kumamoto, Japan; 2 The Global COE, Kumamoto University, Kumamoto, Japan; 3 Dojindo Laboratories, Kumamoto Techno Research Park, Kumamoto, Japan; Wellcome Trust Centre for Stem Cell Research, United Kingdom

## Abstract

To date, CXCR4 and E-cadherin double-positive cells detected by flow cytometry have been used to identify the differentiation of embryonic stem (ES) cells or induced pluripotent stem (iPS) cells into definitive endoderm (DE) lineages. Quantification of DE differentiation from ES/iPS cells by using flow cytometry is a multi-step procedure including dissociation of the cells, antibody reaction, and flow cytometry analysis. To establish a quick assay method for quantification of ES/iPS cell differentiation into the DE without dissociating the cells, we examined whether secreted Cerberus1 (Cer1) protein could be used as a marker. Cer1 is a secreted protein expressed first in the anterior visceral endoderm and then in the DE. The amount of Cer1 secreted correlated with the proportion of CXCR4+/E-Cadherin+ cells that differentiated from mouse ES cells. In addition, we found that human iPS cell-derived DE also expressed the secreted CER1 and that the expression level correlated with the proportion of SOX17+/FOXA2+ cells present. Taken together, these results show that Cer1 (or CER1) serves as a good marker for quantification of DE differentiation of mouse and human ES/iPS cells.

## Introduction

Embryonic stem (ES) cells are derived from a pluripotent inner cell mass, which can be cultured indefinitely in an undifferentiated state and can be differentiated into most cell types in an organism. Therefore, ES cells have been proposed as a source of surrogate cells for use in regenerative medicine.

The definitive endoderm (DE) gives rise to the gastrointestinal organs, such as stomach, pancreas, liver, and intestine. The gastrointestinal organs are of great importance in their therapeutic aspects. Studies of ES cells have demonstrated that ES cell differentiation recapitulates early signaling events of differentiation into the 3 germ layers. Recent progress has identified several germ layer-specific markers of the early DE. Sox17 (Sry-box–containing gene 17), which encodes an endodermal HMG (high mobility group)-box transcription factor, is a DE-specific marker [1]. CXCR4 (C-X-C chemokine receptor type 4), which is expressed in the mesoderm, is also expressed in the DE and is widely used in combination with E-cadherin for the prospective isolation of embryonic or ES cell-derived DE cells [2]. Our group previously identified DAF1 (decay accelerating factor)/CD55 as a novel DE marker [3]. Yasunaga et al., reported the use of the Sox17 promoter to drive the expression of the surface antigen-GFP (green fluorescent protein) fusion protein, which genetically marked the DE with GFP.

Cerberus1 (Cer1; also known as Cerberus like 1 [Cerl1] or Cerberus related gene [Cerr1]) is a secreted protein, which belongs to the cysteine knot superfamily and includes TGF (transforming growth factor) βs and BMPs (bone morphogenetic proteins). Cer1 is first expressed in the anterior visceral endoderm at E6.5 and at E7.0 in the distal visceral endoderm and the definitive endoderm, which emanates from the anterior portion of the primitive streak. Cer1 is expressed in the anterior DE at E7.5 and is expressed in the foregut at the headfold stage. Later, Cer1 is expressed in a limited region in the somatic mesoderm, the pre-somitic mesoderm, and the presumptive foregut endoderm. Cer1 belongs to the Cer/Dan gene family, which contains the secreted antagonists of Nodal, Wnt, or BMP signaling pathways, and plays an important role in regulating these signals [4] [5] [6] [7] [8] [9].

We previously established a procedure to induce ES cells to sequentially differentiate into the mesendoderm, DE, and, finally, regional specific definitive endodermal tissues *in vitro* in a manner that mimics early embryonic inductive events *in vivo* by culturing ES cells on a monolayer of M15 cells [10] [11]. This M15 monolayer culture procedure turned out to be useful not only in directing DE lineages, but also in directing the ES cells to the ectoderm and mesoderm lineages upon altering the culture conditions [12]. We performed gene array analysis of the ES cell-derived lineage-specific progenitors and demonstrated that genes enriched in each cell population are expressed in the normal embryos in a coordinated temporal–spatial fashion [3] [13]. Murine *Cerberus 1* (*Cer1*) is one of the genes that was up-regulated greater than 5-fold in both E-Cadherin+/CXCR4+ DE- and E-Cadherin+/DAF1+ DE-derived from ES cells. Its expression persisted in the DE in early embryos and, therefore, might serve as a good marker for detecting differentiation to DE. Here, to enable quick identification and quantification of the DE in whole ES cell cultures, we established ELISA systems to measure the amount of the secreted protein of mouse *Cer1* and human *CER1*.

## Materials and Methods

### Cell Lines

The ES cell line, SK7 [10], containing a *Pdx1* promoter-driven GFP reporter transgene, was cultured and differentiated as previously described [11] [12]. A mouse iPS cell line (20D17) [14] and a mouse ES cell line (EB3) [15] were also used for endoderm differentiation. The mesonephric cell line M15 [16] was kindly provided by Dr. T. Noce (Mitsubishi Kagaku Institute of Life Science, Tokyo, Japan) and Dr. M. Rassoulzadegan (University of Nice-Sophia Antipolis, Antipolis, France) and is available from the European Collection of Cell Cultures (ECACC 95102517). M15 cells were treated with mitomycin C (Sigma) and were used as previously described [10] [11] [12]. Use of the human ES cells was approved by the Kumamoto University Institutional Review Board and followed the hES cell guidelines of the Japanese government. Undifferentiated human ES cells (khES3) [17] and iPS cells (201B7 and 253G1) [18] were maintained as described [11].

### Differentiation of ES and iPS Cells

For definitive endoderm (DE) differentiation, mouse ES/iPS cells were cultured on M15 cells with added recombinant human activin-A at 10 ng/ml (R&D Systems, Inc) and/or human bFGF at 5 ng/ml (Peprotech) for 3–7 d, as indicated. They were subsequently analyzed using flow cytometry to assay for DE or Cer1 expression [10]. Human ES/iPS cells were cultured on a fibronectin- (Sigma) coated plate in RPMI-1640 (Invitrogen) supplemented with activin-A (100 ng/ml) and a B27 supplement without insulin (2%; Invitrogen) for 6 d [11] [19]. For re-plating of the DE cells, the cells were first dissociated with 0.25% trypsin-EDTA (Invitrogen) and then plated with a 10% FBS medium at concentrations of 0.25 × 10^5^, 0.5 × 10^5^, or 1.0 × 10^5^ cells/well on a matrigel (BD) pre-coated 96-well plate. For neuroectoderm differentiation, ES cells were cultured on M15 cells in a differentiation medium supplemented with 10 µM SB431542 (a TGFβ inhibitor) (Calbiochem, San Diego) [12]. For mesoderm differentiation, mouse ES cells were cultured on M15 cells in a differentiation medium supplemented with 25 ng/ml BMP7 (R&D Systems, Inc.) [12]. Human iPS cells were grown using Stemline II serum-free medium (Sigma) supplemented with 50 ng/mL BMP4 (Peprotech) and ITS (Invitrogen) [20]. For hepatic differentiation of mouse ES cells and human iPS cells, differentiation were done as described [11] [21].

### Real-time and Semiquantitative Reverse Transcription-polymerase Chain Reaction (RT-PCR) Analysis

RNA was extracted from ES cells using the TRI Reagent (Sigma) or RNeasy micro-kit (Qiagen) and then treated with DNase (Sigma). Three micrograms of RNA was reverse-transcribed using MMLV reverse transcriptase (Toyobo) and oligo-dT primers (Toyobo). The primer sequences were as follows: *Cer1* forward 5′-GTCCAGGCTTGGAAGATTC-3′and reverse 5′-AGGGCACAGTCCTGCAGGTC-3′; *Sox17* forward 5′-GAACAGTTGA- GGGGCTACAC-3′ and reverse 5′-GTTTAGGGTTTCTTAGATGC-3′; *Foxa2* forward 5′-TGGTCACTGGGGACAAGGGAA-3′ and reverse 5′-GCAACAACAGCAATAGAG- AAC-3′; *Flk1* forward 5′-CACCTGGCACTCTCCACCTTC-3′ and reverse 5′-GATTTCATCCCACTACCGAAAG-3′; *Zic1* forward 5′-TCGTGTCTCCCACAAC- AGAC-3′ and reverse 5′-GGGGTGTCTCGAGTTCAGG-3′; Human *GAPDH* forward 5′-CGAGATCCCTCCAAAATCAA-3′ and reverse 5′-CATGAGTCCTTCCACGATACC- AA-3′; Human *SOX17* forward 5′-ACTGCAACTATCCTGACGTG-3′ and reverse 5′-AGGAAATGGAGGAAGCTGTT-3′; and Human *CER1* forward 5′-ACAGTGCCCTTCA-GCCAGACT-3′ and reverse 5′-ACAACTACTTTTTCACAGCCTTCGT-3′.

The PCR conditions for each cycle were: (1) denaturation at 96°C for 30 s, (2) annealing at 60°C for 2 s, and (3) extension at 72°C for 45 s. RT-PCR products were separated using 5% non-denaturing polyacrylamide gel electrophoresis, stained with SYBR Green I (Molecular Probes), and visualized using a Gel Logic 200 Imaging System (Kodak).

### Immunocytochemical Analysis

Differentiated ES cells were fixed in 4% paraformaldehyde in phosphate-buffered saline (PBS) for 30 min at room temperature, permeabilized with 0.1% Triton-X100 (Nacalai Tesque) in PBS, blocked with 20% Blocking One (Nacalai Tesque), and then incubated with a diluted antibody in 20% Blocking One in PBST (0.1% Tween-20 in PBS) for 16 h at 4°C. After washing with PBST, cells were incubated with a secondary antibody and 6-diamidino-2-phenylindole (DAPI) (Roche Diagnostics) in PBST for 2 h. The following antibodies were used as primary antibodies: rabbit anti-AFP (A0008, Dako), rat anti-mouse Cer1 (MAB1986, R&D Systems, Inc), goat anti-mouse Cer1 (AF1986, R&D Systems, Inc), mouse anti-human CER1 (MAB1075, R&D Systems, Inc), goat anti-human CER1 (AF1075, R&D Systems, Inc), rabbit anti-Foxa2 (70–633, Millipore), goat anti-T (AFP2085, R&D Systems, Inc), and Alexa 488-,568 or 633-conjugated secondary antibodies (Molecular Probes).

### Flow Cytometry Analysis

Cells were dissociated with a cell dissociation buffer (Invitrogen), adjusted to 1 × 10^6^ cells/50 µl, and stained with their corresponding antibodies. The following antibodies were used: biotin- or Alexa 488-conjugated anti-E-cadherin monoclonal antibody (mAb) ECCD2, and biotin- or phycoerythrin (PE)-conjugated anti-CXCR4 mAb 2B11 (BD Pharmingen). The stained cells were analyzed using a FACS Canto (BD Pharmingen). Data were recorded using the BD FACSDiva Software program (BD Pharmingen) and analyzed using the Flowjo program (Tree Star).

### Western Blot Analysis

Cells and supernatants were lysed in a sample buffer (50 mM Tris-HCl [pH 6.8], 5% glycerol, and 1% SDS (sodium dodecyl sulfate) and boiled for 3 min at 95°C. The samples were separated on SDS-PAGE, transferred onto a PVDF membrane (Immobilon; Millipore, Bedford, MA) and detected with a goat anti-mouse Cer1 or goat anti-human CER1 antibody. Horseradish peroxidase-conjugated (Santa Cruz Biotechnology) antibodies were used as secondary antibodies (at 1∶20,000 dilution). The chemiluminescent signals were detected with ECL Plus (GE Healthcare) and scanned by ImageQuant LAS 4000 (GE Healthcare).

### Immunoprecipitation

The culture supernatants were incubated with a binding buffer (50 mM KCl, 5 mM MgCl_2_, 1 mM EDTA, 20 mM Tris-HCl, pH 8.0, and 0.1% Triton X-100), prewashed protein G-Sepharose beads (GE Healthcare), and goat anti-mouse Cer1 or goat anti-human CER1 antibody at 4°C for 16 h. Then, the precipitated proteins were subjected to western blot analysis.

### ELISA for Detecting Murine Cer1 and Human CER1

A goat anti-mouse Cer1 or goat anti-human CER1 antibody at 1 µg/ml in PBS was immobilized onto 96-well plates (Nunc, MaxiSorp) for 16 h at 4°C. After washing with 0.05% Tween-20 in PBS (PBST), the plate was blocked with blocking one (Nacalai) in PBST. Standards or samples were added and allowed to react with the immobilized antibody at 25°C for 60–120 min. After washing with PBST, horseradish peroxidase-conjugated rat anti-mouse Cer1 or mouse anti-human CER1 antibody, prepared by using an easy labeling kit (Dojindo), were added and subsequently incubated at 25°C for 60 min. After washing with PBST, a TMB substrate solution (Kirkegaard & Perry Laboratories) was added to each well. Enzyme activity was determined by measuring the absorbance at 450 nm after termination of the reaction by the addition of equal 0.1 M sulfuric acid.

### Preparation of Standard Human Cerberus Protein

A cDNA fragment of human *CER1* (nt. 1–804; NP_005445.1aa: 1–267) [22] was sub-cloned into a pCold I vector (TAKARA) with the His-tag sequence in the *N*-terminal. The CER1 recombinant protein was produced in the *Escherichia coli* strain Rosetta-gami (DE3) (Novagen). The bacteria were incubated overnight in Luria-Bertani (LB) medium at 37°C and cooled on ice for 15 min. Protein production was promoted in 0.1 mM isopropyl-*β*-D-thiogalactopyranoside (IPTG) and incubated at 16°C for 16 h. The bacterial cells, collected by centrifugation, were immediately frozen in liquid N_2_ and stored at −70°C until use. The bacteria cells were sonicated on ice containing a protease inhibitor cocktail (Nacalai). The supernatant was precipitated with a 30% saturation of ammonium sulfate on an ice bath. The precipitate was re-dissolved in a Tris buffer containing 5 mM imidazole and a protease inhibitor cocktail. It was then poured onto a Ni Sepharose 6 Fast Flow (GE Healthcare) medium, pre-equilibrated with the same buffer, and gently swirled at 4°C for 16 h. After washing with the same buffer containing 100 mM imidazole, hCER1 was eluted in a stepwise manner (150–250 mM imidazole). The eluted hCER1 protein was concentrated with a filtration system (PALL), quantified by a Protein Quantification kit (Dojindo), and analyzed with 12.5% SDS-PAGE. The protein was stained with Coomassie Brilliant Blue (CBB) to show a single band of recombinant human CER1 protein.

## Results

### Murine Cerberus 1 is a Secreted Protein Expressed in the Definitive Endoderm Derived from Mouse ES Cells

**Figure 1:**
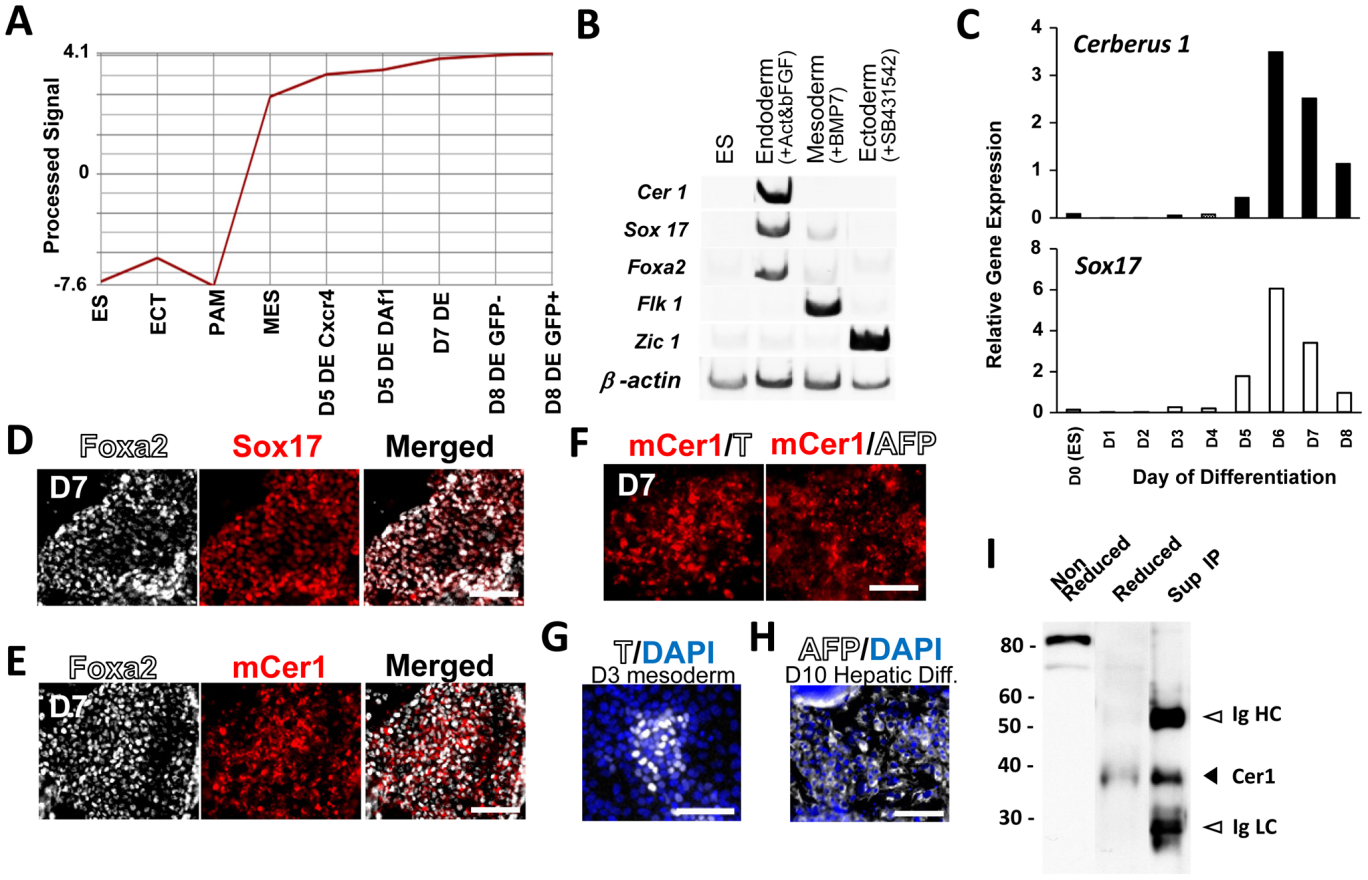
Murine Cerberus1 is a secreted protein expressed in the mouse ES cell-derived definitive endoderm. (A) Cer1 is up-regulated in the definitive endoderm (DE) population and assayed by expression profiling analysis. Y axis: Cer1 expression level; X axis: different germ layer cells and populations on different differentiation days (D) derived from ES cells. ES cells were differentiated into the 3 germ layers under different conditions [12] [25]. ES cell-derived differentiated cells of the ectoderm (ECT) (SSEA1−/Flk1−/PGFRα-), mesoderm (MES) (E-cadherin+/PDGFRα+), paraxial mesoderm (PAM) (E-cadherin−/PDGFRα+/Flk1−), and DE (E-cadherin+/CXCR4+ populations: D5 DE Cxcr4, D7 DE; and E-cadherin+^/^Daf1+ populations: D5 DE Daf1 [3] at D5 and D7) were prospectively isolated by the expression of specific cell surface antigens using flow cytometry. DE cells at D8 were further subdivided into *Pdx1*/GFP-negative and -positive populations (D8 DE GFP- and GFP+). (B) Semiquantitative RT-PCR of *Cer1*, *Sox17*, *Foxa2*, *Flk1*, and *Zic1* in ES cell-derived differentiated cells treated with activin A 10 ng/ml and bFGF 5 ng/ml, BMP 7 25 ng/ml, or SB432542 10 µM. (C) Time-dependent expression of *Cer1* and *Sox17* detected by real-time PCR. (D, E, F) Immunocytochemical analysis. At D7, almost all Foxa2+ cells are co-stained for Sox17 (E) Cer1 is expressed in the Foxa2+ cells (F) Cer1 positive cells do not express T or AFP at D7. (G) T is expressed in mouse ES derived mesoderm cells. (H) AFP is expressed mouse ES derived hepatic cells (I) Western blot analysis for Cer1 with non-reduced/reduced whole cell extract of differentiated ES cells and immunoprecipitation of the supernatant (Sup IP). Scale bar = 100 µM.

Cer1 was one of the genes that were specifically up-regulated into the mesendoderm and CXCR4+/E-cadherin+ or DAF1+/E-cadherin+ DE populations at differentiation days (D) 5, 7, and 8 compared to that in undifferentiated ES cells, ectoderm (ECT), or paraxial mesoderm (PAM) in our gene array analysis (Fig. 1A) [3]. *Cer1* expression was maintained in the *Pdx1*/GFP-positive or negative populations (Fig. 1A; D8 DE GFP+ and D8 DE GFP^–^). *Cer1* was also expressed in the DE. To confirm *Cer1* expression in the DE, ES cells were selected to undergo differentiation in the cells of the 3 germ layers. Semiquantitative RT-PCR analysis revealed that, when ES cells underwent endoderm differentiation through the addition of activin A and bFGF, *Cer1* expression was up-regulated in conjunction with the expression of DE markers *Foxa2* and *Sox17*. This was not observed when ES cells were differentiated into the mesoderm, marked by *Flk1* expression triggered by BMP7, or neuronal ectoderm differentiation, marked by *Zic1* expression, when added with SB431542, an inhibitor for TGFβ signaling (Fig. 1B). Time-dependent expression of *Cer1* detected by real-time PCR revealed that *Cer1* expression reached peak differentiation on D6, which then decreased on D7. The expression of *Sox17* [1], a DE marker, showed a similar pattern (Fig. 1C). Immunocytochemical analysis using an anti-Cer1 polyclonal antibody confirmed that Cer1 was expressed in Foxa2+/Sox17+ DE cells. Furthermore, these Cer1+ cells did not express T, a mesoderm marker, or a visceral endoderm marker AFP at D7 under this condition (Fig. 1E–F). T or AFP was expressed in mouse ES cell-derived mesoderm or hepatic cells (Figure 1G, H).

We then confirmed the expression of the Cer1 protein in the differentiated ES cells. The crude lysate from the ES cells derived from DE were extracted and subjected to a western blot analysis. Under non-reduced and reduced conditions, Cer1 was detected as an 80-kDa or a 39-kDa protein, respectively, indicating that Cer1 exists as a dimer, which has a slightly larger molecular weight than the 32 kDa previously reported [5]. We then asked whether we could detect the secreted Cer1 protein. Secreted Cer1 in the culture supernatant was immunoprecipitated with a polyclonal antibody against Cer1. Western blot analysis revealed that Cer1 was precipitated as a 39-kDa protein (Fig. 1I, arrow head).

These results indicate that the Cer1 protein is expressed and secreted upon the differentiation of ES cells into DE.

### Establishment of an ELISA System for Quantification of the Secreted Mouse Cer1 Protein

**Figure 2:**
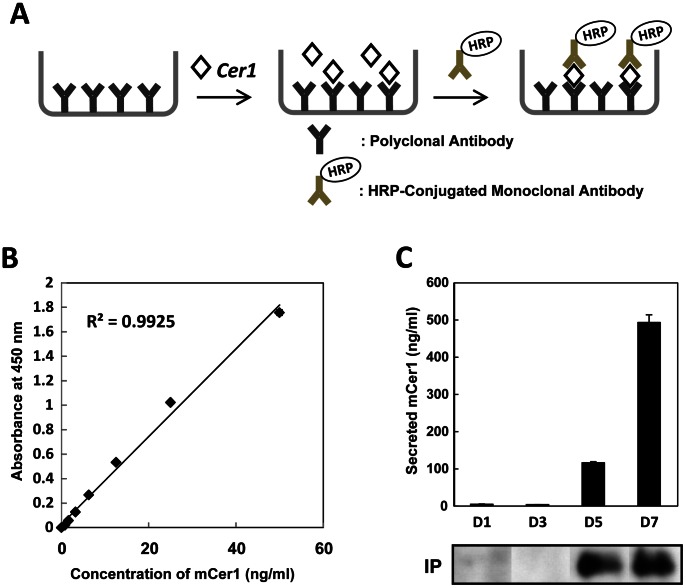
Establishment of an ELISA system for quantification of the secreted mouse Cer1 protein. (A) Schematic drawing of the ELISA system. (B) Standard curve using purified Cer1. (C) ELISA assays for the secreted Cer1 protein at differentiation days (D) 1, D3, D5, or D7. The supernatant was sampled 48 h (lower panel) after replacement with fresh media. Time-dependent expressions of Cer1 in the supernatant, detected by immunoprecipitation, are also shown (bottom panels).

To quantify the secreted Cer1 protein, we established an ELISA assay system. Fig. 2 shows a schematic drawing of the ELISA assay system. The polyclonal anti-Cer1 antibody was immobilized onto 96-well plates. Then, serial dilutions of standard Cer1 samples were added. The HRP conjugated anti-Cer1 monoclonal antibody was used to detect Cer1, and the substrate was added to visualize HRP activity (Fig. 2A). The Cer1 standard curve showed a good correlation between HRP activities and concentrations of the Cer1 protein (Fig. 2B).

We then used this ELISA system to quantify the secreted Cer1 protein in the differentiated ES cells (Fig. 2C). ES cells were differentiated into the DE lineage. Culture supernatants from differentiation D1 to D7 were assayed for ELISA and immunoprecipitation, which was followed by a western blot analysis. Secreted Cer1 was detectable from differentiation on D5, which further increased on D7 (Fig. 2C, upper panel). Immunoprecipitation analysis confirmed that the secreted Cer1 protein was present in the supernatant (Fig. 2C, lower panel).

### The Amount of Secreted Cer1 Protein Correlates with the Proportion of DE Derived from Mouse ES/iPS Cells

**Figure 3:**
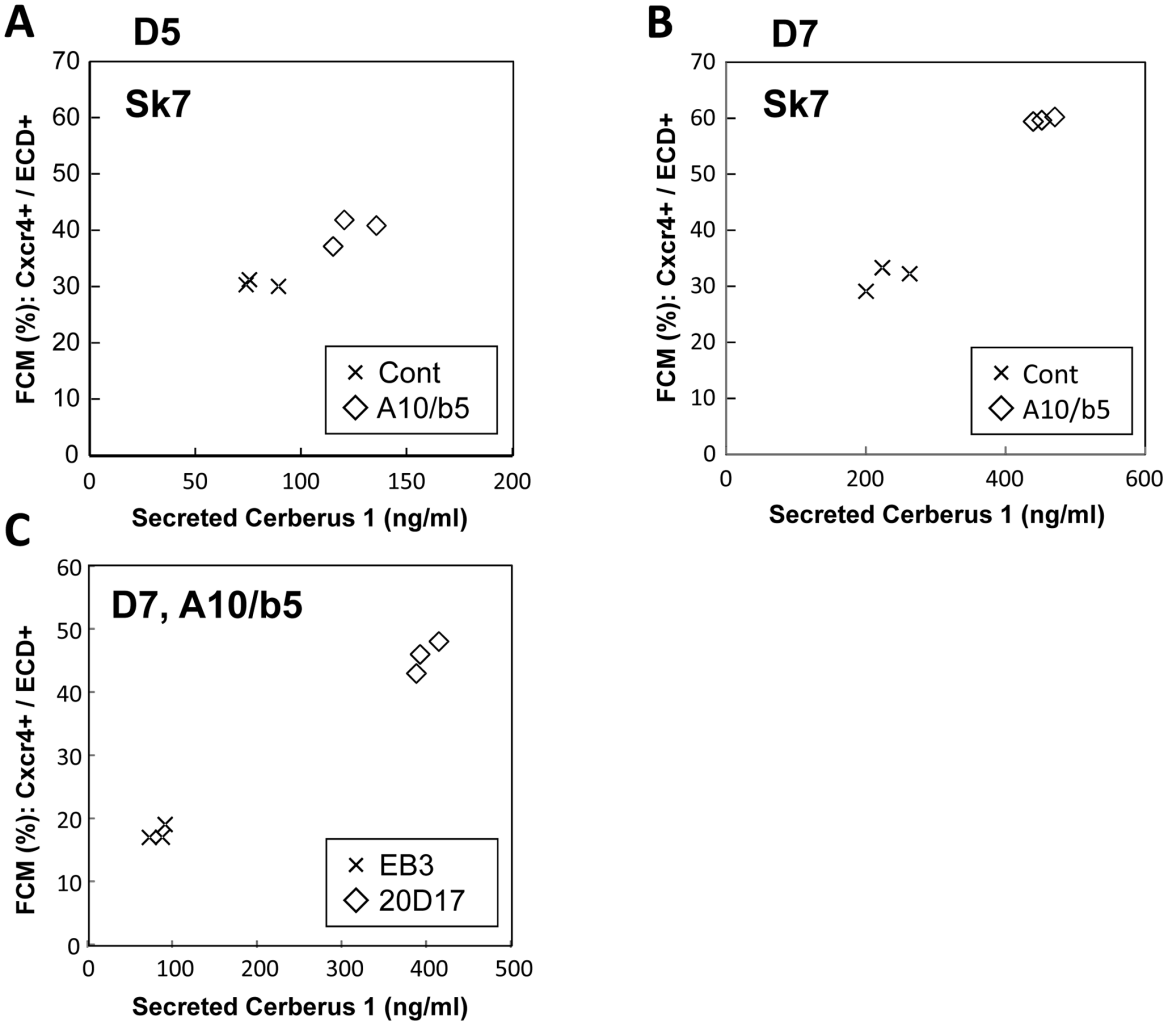
Figure 3. The amount of secreted Cer1 correlated with the proportion of DE differentiation of mouse ES/iPS cells. (A, B) The proportion of DE cells assayed by flow cytometry correlated with the amount of the Cer1 protein secretions assayed on day (D) 5 (A) or D7 (B) of differentiation using the SK7 ES cell line. Different growth factors were added for obtaining different degrees of definitive endoderm (DE) differentiation. A10, Activin 10 ng/ml; b5, bFGF 5 ng/ml; control (Cont), no growth factors. (C) The proportion of DE assayed by flow cytometry correlated with the level of the secreted mouse Cer1 assayed on D7 of differentiation added with activin and bFGF using the EB3 ES and 20D17 iPS cell lines.

To test if the secreted Cer1 protein could be used to assess the proportion of the DE, we examined the correlation of the amount of secreted Cer1 with flow cytometry analyses of the DE population. ES cells differentiated into the DE with the addition of activin A and bFGF, which gave rise to different proportions of the DE. The cells were then subjected to flow cytometry analysis for Cxcr4+/E-cadherin+ DE cells or an ELISA assay for quantification of the amount of the Cer1 protein secreted. Culture supernatants were collected on differentiation D5 or D7, 48 h after replacement with fresh media. At the same time, cells were analyzed by flow cytometry on D5 or D7. The amount of secreted Cer1 protein was higher on D7 than on D5 (Fig. 2C; Fig. 3), while the amount of secreted Cer1 correlated with the proportion of Cxcr4+/Ecadherin+ cells on D5 and D7 of the DE, respectively (Fig. 3A, B). We also confirmed these results with a mouse ES cell line (EB3) and a mouse iPS cell line (20D17). Cer1 secretion correlated with the proportion of the Cxcr4+/Ecadherin+ DE cells at D7 in these cell lines, which showed a different propensity for differentiation into the DE (Fig. 3C). Therefore, measuring the secreted Cer1 protein on the same differentiation day was useful to quantify DE population size from the ES/iPS cells.

### Establishment of an ELISA System for Quantification of the Secreted Human CER1 Protein

**Figure 4:**
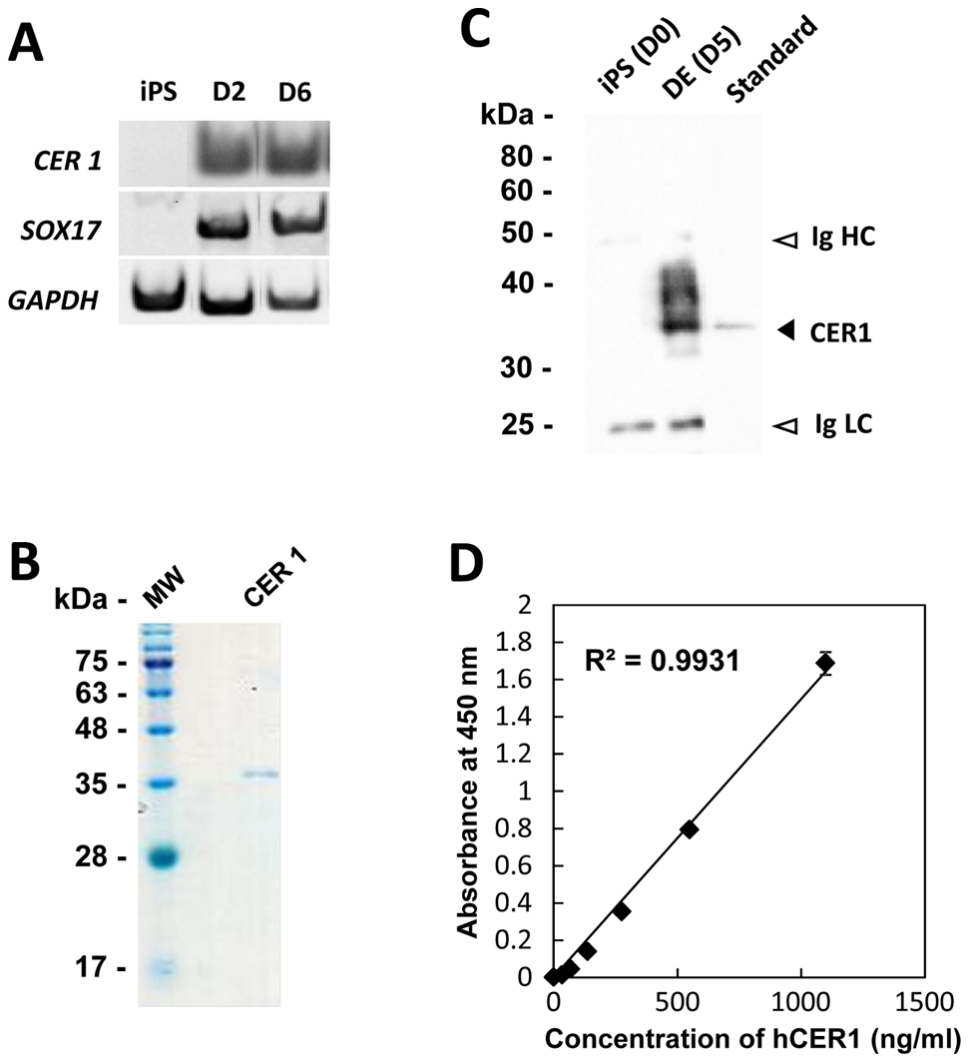
Establishment of an ELISA system for quantification of the secreted human Cer1 protein. (A) Time-dependent expressions of *CER1* and *SOX17* transcripts on differentiation day 2 (D2) and D6 of the human iPS cell line, 201B7 assayed by RT-PCR are shown. (B) SDS-PAGE analysis of recombinant human CER1 protein. (C) Western blot analysis of recombinant human CER1 (standard) and immunoprecipitation of the supernatant using undifferentiated iPS cells (D0) and differentiated DE (D5) medium. (D) Standard curve using recombinant human CER1.

To examine if the ELISA system (see above) could be applied to human ES/iPS cells, we differentiated a human iPS (hiPS) cell line (201B7) [18] into the DE. CER1 expression was detected on D2 and was coordinated with SOX17 expression, as detected by semi-quantitative RT-PCR analysis (Fig. 4A). We prepared the recombinant human CER1 protein for use as the standard protein for the ELISA assay. A His-tagged recombinant human CER1 protein was over-expressed in the bacteria and Ni-affinity chromatography was purified into a single band, as revealed by 12.5% SDS-PAGE and CBB staining (Fig. 4B). Immunoprecipitation followed by western blot analysis of the culture supernatant from the hiPS cell-derived DE on D5 confirmed the expression of CER1 (Fig. 4C). The recombinant CER1 was then used as the standard for the ELISA assay to quantify the amount of CER1 (Fig. 4D).

### CER 1 is a Secreted Protein Expressed in the DE Derived from Human ES/iPS Cells

**Figure 5:**
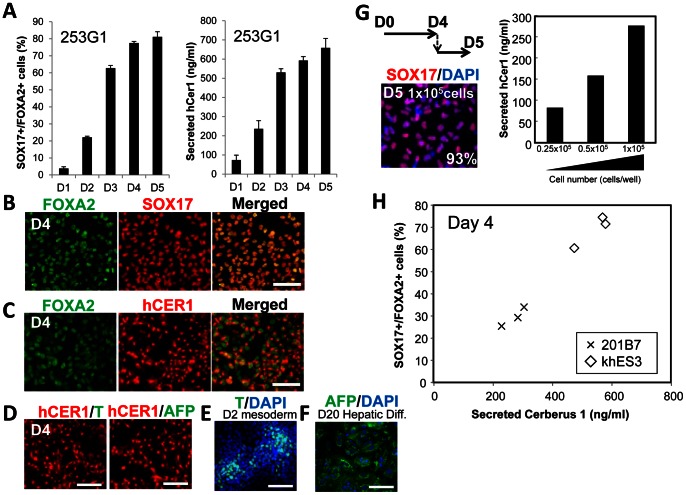
The amount of secreted CER1 correlated with the DE differentiation of human iPS cells. (A) The supernatant was sampled 24 h after replacement with fresh media on differentiation days 1 to 5 (D1 to D5) of the human iPS cell line (253G1). ELISA assays were performed to measure secreted CER1, and quantified by comparison with the standard CER1. The proportion of SOX17+/FOXA2+ DE was calculated from the immunocytochemical analysis results. Values represent the mean ± S.E.M. (N = 3). (B) At D4, approximately all the FOXA2+ cells co-expressed SOX17. (C) Human CER1 staining was observed in approximately all the FOXA2+ cells (D) CER1+ cells did not express T or AFP at D4 in our differentiation system. (E) T is expressed in human iPS cell-derived mesoderm cells. (F) AFP is expressed in human iPS cell-derived hepatic cells (G) ELISA and immunocytechemical analysis of the re-plated DE cells. (H) The proportion of SOX17+/FOXA2+ DE correlated with human CER1 secretion assayed on D4 of differentiation using the 201B7 human iPS cell line and khES3 human ES cell line. Scale bar = 100 µM.

Next, we examined the correlation of the amount of secreted CER1 with immunocytochemical analyses of the DE population. Since the 201B7 hiPS cell line showed a low propensity for differentiation into the DE, we then used another hiPS cell line (253G1) [18]. 253G1 cells were differentiated into the DE and subjected to immunocytochemical analysis for SOX17+^/^FOXA2+ cells or ELISA assay to measure the amount of CER1 secreted. The secreted CER1 protein amount correlated with the amount of SOX17+/FOXA2+ cells (Fig. 5A). Similar to observations made using mouse Cer1, human CER1 was expressed in SOX17+/FOXA2+ DE cells (Fig. 5B, C) and did not overlap with T or AFP expression (Fig. 5D). T or AFP was expressed in human iPS cell-derived mesoderm or hepatic cells (Figure 1E, F). We re-plated cells in several cell densities on D4 (Fig. 5G). One day after re-plating (D5 equivalent), an ELISA assay and immunocytochemical analyses were performed. As a result, more than 90% of the cells were SOX17+ DE cells, and the amount of human CER1 increased depending on the cell numbers seeded (Fig. 5G). The ELISA assay of 201B7 and human ES cell line (khES3) revealed that human CER1 secretion correlated with the amount of SOX17+/FOXA2+ DE cells in both human iPS and ES cells (Fig. 5H). Taken together, the presented ELISA assay system enables the quantification of the amount of CER1 protein secreted and the proportion of DE differentiation of human ES/iPS cells.

## Discussion

Here, we described the development of an ELISA assay system for detecting murine Cer1 protein or human CER1 secreted from the DE cells derived from mouse ES cells or human iPS cells. Quantification of the Cer1 protein using the ELISA assay system revealed an approximate correlation with the amount of DE cells, thereby indicating that the Cer1 ELISA system could be used for quick quantification of the number of DE cells derived from mouse or human pluripotent cells.

In our mouse ES cell differentiation system, where the DE and its derivatives could be induced, we observed the expression of Cer1 in mesendoderm cells, which were then up-regulated in D5 DE. High expression of Cer1 was maintained through D7 and D8 DE in the Pdx1-positive and Pdx1-negative cells. Cer1 was also expressed in the DAF1+/E-Cadherin+ DE [3]. This result is in agreement with our previous results that DAF1+/E-Cadherin+ is a good marker to detect the late DE, where CXCR4 expression decreased rapidly after establishment of the DE. *Cer1* transcript expression seemed to come to a peak before the secreted Cer1 protein. This may be a reflection of the fact that the accumulation of the Cer1 protein takes time, and there is a time lag between *Cer1* transcription and the secreted protein expression.

Cer1 is a secreted protein and is reported to be modified by *N*-glycosylation [5]. Our results suggested that the Cer1 expressed in mouse ES cells and human iPS cells are also *N*-glycosylated. Cer1 is known to primarily be expressed in early mouse embryos, first in the anterior visceral endoderm and anterior DE where it functions to bind and block Nodal and BMP signaling but not Wnt signaling [6] [23] [24]. Cer1 expression in the anterior DE disappeared at later stages. Therefore, Cer1 is a marker for anterior DE, but not for the entire DE, in a stage dependent manner. Although Cer1 is reported to be expressed later in the mesoderm [5], we did not observe Cer1 expression in our ES cell differentiation system, where mesoderm cells could be induced by adding BMP (Fig. 1A, B) [12]. This might be due to the limited expression region of the Cer1 in the mesoderm and the low expression of Cer1, which might not be detected in this narrow window of time. In both mouse and human differentiated cells, Cer1 was expressed in Sox17+/Foxa2+ cells. These Cer1+ cells did not express T or AFP under our differentiation conditions (Fig. 1D–F and Fig. 5B–D). However, since Cer1 is a marker for anterior DE, but not for the entire DE and is expressed in the mesoderm or visceral endoderm, we should be aware that the amount of Cer1 is not always proportional to the total amount of DE in the various conditions of differentiation. Therefore, confirmation using other markers for DE, or differentiation using another protocol, is recommended.

Taken together, our present ELISA system for measuring the amount of mouse Cer1 or human CER1 secreted allows quick quantification of the DE in living ES/iPS cells. Secreted Cer1 or CER1 protein levels could be used as a parameter for comparing the propensity of differentiation into the DE among different ES/iPS cell lines. An ELISA assay for detecting Cer1 or CER1 secretions offers an easy and quick analysis and could be applied to large-scale analyses. It is useful for monitoring differentiation of ES/iPS cells, particularly in experiments such as chemical screenings for drugs that potentiate subsequent differentiation of the DE lineages.
